# Efficacy and safety of Naoxintong capsule for treating chronic stable angina: study protocol for a randomized controlled trial

**DOI:** 10.1186/s13063-021-05264-y

**Published:** 2021-05-10

**Authors:** Gao Huanjia, Cai Hairong, Zhuang Jieqin, Dai Xingzhen, Fu Xue, Zhang Weizhang, Chen Bojun

**Affiliations:** 1 The Second Clinical College of Guangzhou University of Chinese Medicine, Guangzhou, 510405 Guangdong Province China; 2 The Second Affiliated Hospital of Guangzhou University of Traditional Chinese Medicine, Guangzhou, 510006 Guangdong Province China

**Keywords:** Chronic stable angina, Naoxintong capsule, Traditional Chinese medicine, Randomized controlled trial

## Abstract

**Background:**

Cardiovascular disease is the leading cause of mortality and morbidity worldwide, Chronic stable angina (CSA) is the main symptom of myocardial ischemia, causes increased risk of major cardiovascular events such as sudden cardiac death and myocardial infarction. Naoxintong (NXT) capsule is a classical traditional Chinese medication used to treat CSA, however, few evidence to support the wide utility of NXT capsule for the treatment of CSA. We design this study to evaluate the efficacy and safety of NXT capsule versus placebo in patients with CSA.

**Methods/design:**

This is a multicenter, randomized, double-blind, placebo-controlled clinical trial. A total of 260 eligible participants will be enrolled. The participants will be randomized assigned in an equal ratio to groups receiving either NXT or placebo for 12 weeks. After a 2-week run-in period, they will receive either NXT or placebo (3 pills, 3 times daily) for 12 weeks. The primary outcome is therapeutic efficacy. Secondary outcome measures include the quantitative score of TCM syndromes, severity grading of angina pectoris, the number of angina pectoris per week, nitroglycerin dosage, score of Seattle angina scale, serum homocysteine, and incidence of cardiovascular events. Safety outcomes and adverse events will be monitored throughout the trial.

**Discussion:**

We designed this study in accordance with principles and regulations issued by the China Food and Drug Administration (CFDA). The results will provide clinical evidence of the efficacy and safety of NXT Capsule in the treatment of CSA.

**Trial registration:**

Chinese Clinical Trial Registry ChiCTR2100044563. Registered on 24 March 2020.

## Background

Cardiovascular disease, despite many advances in its diagnosis and treatment, remains the leading cause of morbidity and mortality worldwide, leading to over 17.3 million deaths per year and perhaps growing to over 23.6 million deaths per year by 2030 [1, 2]. CSA is a major symptomatic presentation in about 50% of patients with coronary heart disease (CHD) [3], which is characterized by chest pain or discomfort caused by a temporary disruption in the flow of blood and oxygen to the heart. CSA always triggered by exertion, rest or sublingual nitroglycerin usually relieves angina within 30 s to several seconds [4, 5]. The goals of treating CSA are to improve quality of life and minimize the risk of cardiovascular events [6]. At present, guideline-directed therapy for CSA includes nitrates, beta-blockers, calcium channel blockers, angiotensin-converting enzyme inhibitors statins, and antiplatelet agents [4]. In addition, lifestyle changes, such as maintaining a healthy weight, consuming a low-fat diet, discontinuing the use of tobacco products, and finding ways to reduce stress are also encouraged [7].

Chinese patent medicines (CPMs) are widely used in China as adjuvant therapies for western medicines. TCM has the advantage of its multi-target and multi-link therapeutic effects and less adverse reactions than western therapies, and also has a long history in treating CSA, NXT is a commercial medicinal product approved by the China Food and Drug Administration which is widely used in the treatment of stroke and coronary heart disease, existing studies have demonstrated NXT can help control angina pain, and NXT combined with anti-anginal medications have been proved effective and safe in the treatment of CSA [8, 9]. NXT is made up of several traditional Chinese medicines which can activate blood circulation and remove blood stasis (Table 1). Although there are some Cochrane reviews that have shown the potential benefit of TCM in treating CHD, the current research is methodologically weak; it is meaningful to accumulate more evidence from high-quality trials to demonstrate the clinical use of Chinese patent medicine. We designed a randomized controlled trial for the purpose of providing high-level evidence for the clinical application of NXT capsule and clarifying the efficacy and safety of NXT capsule in the treatment of patients with CSA through multicenter, randomized, double-blind, placebo-controlled, clinical trial.

**Table 1:** Pharmacological effects of each ingredient in NXT

Ingredient	Pharmacological effects
Red peony root, Salvia miltiorrhiza, Angelica sinensis, Chuanxiong, Peach kernel, Safflower, Caulis spatholepis, Earthworm, Leech	Invigorate the circulation of blood
Astragalus, Oxknee	Boost immunity
Cassia twig, Mulberry twig	Dredging collaterals

## Methods/design

**Fig. 1:**
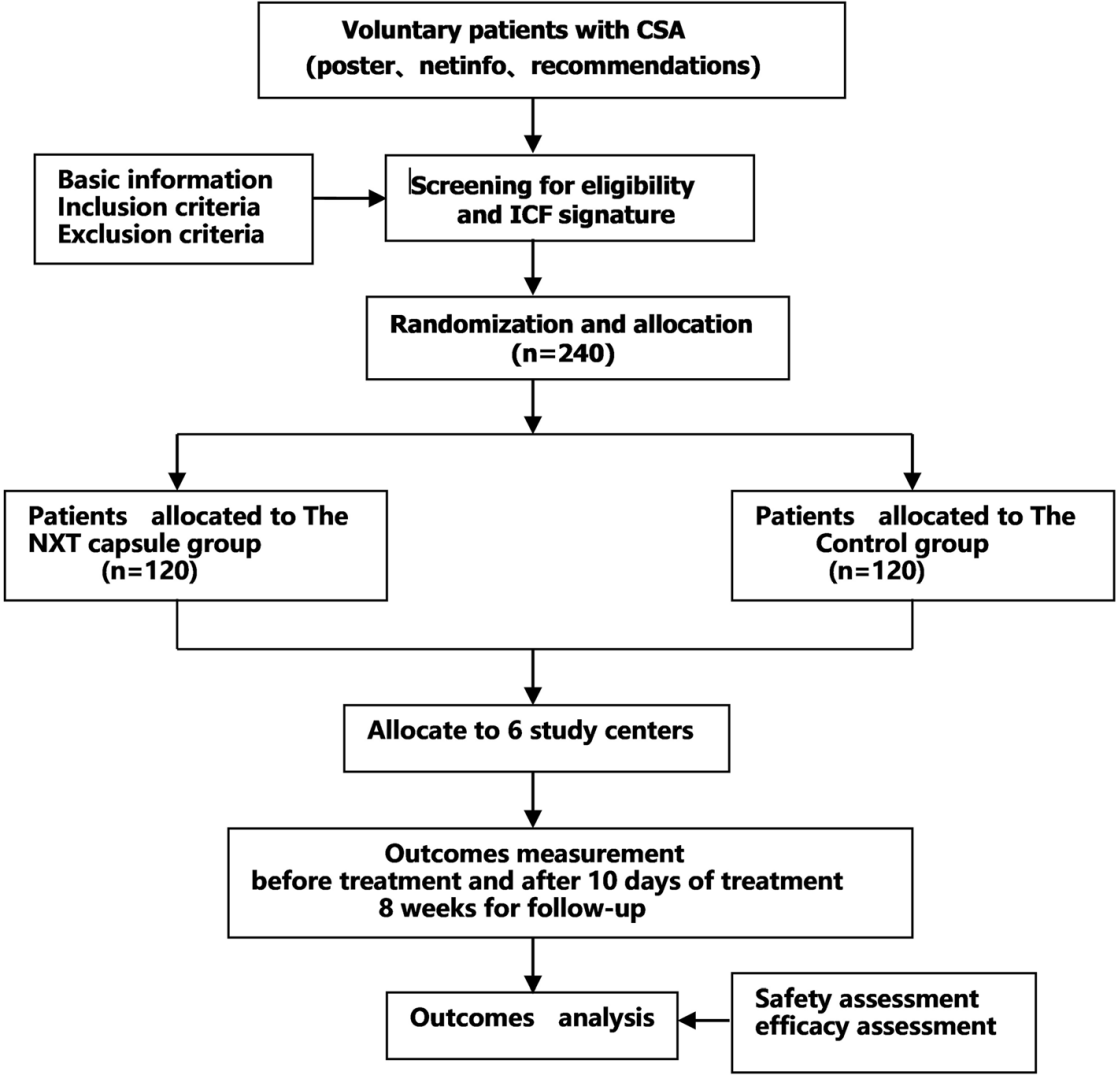
Study flow chart. ICF informed consent form

This study is a multicenter, randomized, double-blind, placebo-controlled, clinical trial. A total of 240 eligible participants will be enrolled. The participants will be randomized assigned in an equal ratio to groups receiving either NXT or placebo for 12 weeks. After a 2-week run-in period, they will receive either NXT or placebo (3 pills, 3 times daily) for 12 weeks. Efficacy and safety data will be obtained during the treatment. A flow chart of the trial is illustrated in Fig. 1.

### Ethics

This trial has been registered in the China Clinical Trial Registry (ChiCTR2000034871). This trial is reported in accordance with the Standard Protocol Items: Recommendations for Intervention Trials (SPIRIT) guidelines [10, 11]. Ethical approval has been obtained from the Ethics Committee of the Guangdong Provincial Hospital of Traditional Chinese Medicine (BF2020-152-01). Participants who meet all of the inclusion criteria will be asked to sign an informed consent before the trial that will contain the details about the trial such as inclusion criteria, exclusion criteria, therapeutic interventions, scheduling, trial benefits, and possible risks of this trial. Participants have the right to leave at any point throughout the trial.

### Participants and recruitment

Participants will be recruited through Internet advertisements and posters in the community and selected hospitals. A total of 240 eligible participants will be recruited in the following six hospitals: (1) Guangdong Provincial Hospital of Traditional Chinese Medicine, (2) The First Affiliated Hospital of Sun Yat-sen University, (3) Zhujiang Hospital, (4) Southern Medical University Hospital, (5) Shenzhen Longgang District Hospital of Traditional Chinese Medicine, and (6) Yangjiang People’s Hospital. The first center will recruit 80 patients, with 32 for each of the remaining centers.

### Eligibility criteria

Eligible participants are those who fulfill all of the listed inclusion criteria and do not have any of the exclusion criteria.

### Diagnostic criteria

#### The diagnostic criteria for CHD

1. A history of myocardial infarction, with or without revascularization (percutaneous coronary intervention (PCI) or coronary artery bypass grafting) treatment;
2. Coronary angiography confirmation or computed tomography coronary angiography confirmation of stenosis greater than 50% of at least 1 major branch of the coronary artery luminal diameter, with or without revascularization;
3. Noninvasive imaging stress test diagnostic of CHD.

A patient meeting at least one of the above criteria is considered to be diagnosed with CHD.

#### The diagnostic criteria for CSA

Diagnostic criteria for CSA were determined according to the Chinese Medical Association’s 2007 Guidelines for the Diagnosis and Treatment of Chronic Stable Angina [12], 2013 ESC guidelines on the management of stable coronary artery disease [13], The classification of angina referred to the Canadian Cardiovascular Society (CSS) Functional Classification of Angina.

#### Inclusion criteria

1. A diagnosis of CHD and the provision of exact imaging information (coronary angiography or computed tomography coronary angiography confirmation of stenosis greater than 50% of at least one major branch of the coronary artery luminal diameter, or nuclear perfusion scan diagnosed as CAD). CCS classification of angina grade II
2. The onset of angina pectoris ≥ 3 months previous and the frequency of angina attack ≥ twice a week
3. Age between 35 and 75, regardless of gender
4. Signed informed consent by participants or surrogates

#### Exclusion criteria

1. Uncontrolled or mismanaged blood pressure and blood glucose. Severe cardiopulmonary insufficiency, or severe arrhythmia (rapid atrial fibrillation and flutter, paroxysmal ventricular tachycardia second degree and greater than a second degree atrioventricular (AV) block). Acute MI in the past 2 months, or has undergone coronary revascularization in the past 12 months.
2. Renal dysfunction, male serum creatinine > 2.5 mg/dL (> 220 μmol/L) or female serum creatinine > 2.0 mg/dL (> 175 μmol/L). Serious liver disease (expression of aminotransferase (ALT) and aspartate aminotransferase (AST) of 1.5 times higher than the normal upper limit).
3. Factors that precluded satisfactory interpretation of the electrocardiogram (ECG) (e.g. digoxin therapy, left bundle branch block, implanted with pacemaker, left ventricular hypertrophy, or electrolyte disturbance).
4. Complications with a serious bone joint disease or other comorbidities that may interfere with the ability to perform required ETT.
5. Patients planning to undergo coronary revascularization during the study period.
6. Patients who might be allergic or are known to be allergic to ingredients of the study drug.
7. pregnant, pregnancy planners, or lactating women.
8. Patients who are allergic to NXT capsule.
9. Substance abuse, alcohol and drug dependence in the last 2 years.
10. Patients who participated in other clinical drug trials within 1 month.

### Withdrawal criteria

The rejection criteria include the following:

1. Patients who experience serious complications or rapid deterioration of the condition throughout the trial.
2. Serious adverse events (AEs) occurring, which would result in treatment being stopped according to the doctors’ decision.
3. Participants with vital deviations in the implementation of the study, such as poor compliance and difficulty in evaluating drug effects
4. Patients who quit this clinical trial voluntarily

### Sample size

The sample size was estimated to treat the symptoms of angina pectoris for 12 weeks as the main effect index according to the statistical requirements. According to the literature, the total effective rate of basic treatment + placebo was 67.5%, and the total effective rate of basic treatment + test drug was estimated to be 84.3. %, let *α* = 0.05, 1-*β* = 0.2, according to the test group: control group = 1:1 set, after calculation, the minimum sample size that meets the statistical requirements is 98.4 cases per group, considering no more than 20% of the shedding rate was 120 cases in each group, a total of 240 cases.

### Randomization and blinding

A total of 240 participants will be randomized in a 1:1 ratio assigned to the NXT group and the control group by the method of using 240 opaque envelopes. Half of them are labeled with the NXT group, and half of them are the control group. After all the envelopes have been sealed, they will be mixed evenly and distributed to each research center. When a sub-center accepts an eligible patient, the baseline information such as the subject’s hospital ID number, name, age, and gender will be recorded. All participants, investigators, and attending physicians will be blinded to the treatment assignments until the research is finished. Only drug administrators and dispensing nurses can open the envelope to check the group allocation and perform the interventions according to the instructions of this study, All investigators, outcome assessors, and data analysts will be blinded to collect and summarize which is only based on a subject’s baseline information until the completion of the visit and analysis.

### Interventions

Eligible patients will be randomly allocated to a treatment group or a control group in an equal ratio. In order to ensure the safety of participants, enrolled patients in both control group and treatment group will receive standard conventional therapies, under the Chinese Guidelines for the Diagnosis and Treatment of CSA (2007) [14]. Routine medications in the trial as follows:

1. antiplatelet agents: aspirin (75–100 mg, once per day) or clopidogrel;
2. Lipid-lowering agents (statins): atorvastatin (10–20 mg, once per day) or simvastatin (20–40 mg, once per day).
3. Anti-angina agents: β-blockers (metoprolol 50–200 mg, once per day, or analogous agents); long-acting nitrates (isosorbide mononitrate 40–60 mg, once per day); or calcium channel blockers (amlodipine 5–20 mg, once per day).

On the basis of conventional western medicine treatment, the experimental group will receive NXT capsule for 12 weeks,3 pills/day, taken orally, after meals; while the control group will receive placebo capsule, the placebo capsule simulator is similar to the NXT capsule, with a comparable appearance, the primary content of the placebo capsule is starch, the control group will receive NXT capsule simulator for 12 weeks, 3 pills/day, taken orally, after meals,

### Efficacy assessment

#### Primary outcome

The primary outcomes of this study include changes of curative effect of angina pectoris symptoms. The number of angina attacks, degree of pain, duration, as well as the dosage of nitroglycerin are used as indicators for scoring. The score ranges from 0 to 15, with higher score indicating a more severe angina (Table 2).

**Table 2:** Measurement items and points of data capture

Study phase	Run-in period		Intervention period		
Time	Visit 1	Visit 2	Visit3	Visit4	Visit5
	−14 ± 2 days	−4 ~ 0 days	4 weeks ± 4 days	8 weeks ± 4 days	12 weeks ± 4 days
Baseline data collection					
Informed consent	×				
Demographic data	×				
Concomitant disease and treatment	×	×	×	×	×
Inclusion/exclusion criteria		×			
Screen index					
Urinary pregnancy test	×				
Safety evaluation					
Vital signs	×	×	×	×	×
Blood and urine routine, stool routine + occult blood, fasting blood glucose		×			×
Liver function (ALT, AST, TBIL) and renal function test (BUN, Cr)		×			×
Coagulation function test		×			×
Blood lipid level		×			×
ECG	×	×			×
Efficiency evaluation					
TCM syndrome score	×	×			×
Scores of angina symptoms	×	×	×	×	×
CCS angina classification	×	×	×	×	×
Number of episodes of AP per week	×	×	×	×	×
Nitroglycerin consumption	×	×	×	×	×
Score of seattle angina scale	×	×	×	×	×
Homocysteine		×			×
Other work					×
Random grouping		×			
Dispense drug	×	×	×	×	
Recovery drug		×	×	×	×
Aes records		×	×	×	×
Evaluate the clinical efficacy and adherence					×

#### Secondary outcomes

1. Therapeutic effect of TCM syndromes: The TCM syndromes scoring system used in this study will follow the guidelines of clinical research on the treatment of coronary heart disease (chest pain) with new Chinese medicine, in which all symptom and sign scores are graded (Table 3). The symptoms of CSA include chest pain, chest tightness, breathlessness, palpitations, mental fatigue, aversion to cold, and cold limbs, lumbar and knee soreness, spontaneous sweating, and insomnia. The score ranges from 0 to 27. Zero indicates asymptomatic, 1 for mild, 2 for moderate, and 3 points for severe. The higher the score, the more severe it is.

**Table 3:** Symptom and sign scores

Symptom or sign	Score			
	Asymptomatic (0)	Mild (1)	Moderate (2)	Severe (3)
Chest pain (angina)	None	Occasionally, Self-medication	Frequently, Obvious when moving	Sustained, Cannot insist on work
Breathless	–	–	–	–
Palpitations	–	–	–	–
Chest tightness	–	–	–	–
Mental fatigue	–	–	–	–
Aversion to cold and cold limbs	–	–	Frequently Need to add clothes	Severe Cannot ease when adding clothes
Lumber and knee soreness	–	–	–	–
Spontaneous sweating	–	Occasionally, Aggravation when moving	Frequently When a little activity	Severe, When no movement
Insomnia	–	Mild Difficult to sleep, but hardly affected	Moderate Cannot sleep for hours	Severe, Cannot sleep all night

(2) Grading changes of the severity of angina pectoris: According to CCS Angina Severity Classification Standard. I: General physical activity does not cause angina pectoris, angina with strenuous, rapid, or prolonged exertion. II: Slight limitation of ordinary activity, angina when walking upstairs briskly, or walking on a cold or windy day. III: marked limitation, angina when walking at a normal pace up flight of stairs, or walking 1–2 blocks distance. IV: Angina on minimal exertion or even at rest.
(3) Frequency of angina pectoris episodes per week.
(4) The dosage of nitroglycerin;
(5) Seattle Angina Questionnaire (SAQ): The SAQ quantifies patients’ physical limitations caused by angina, the frequency of and recent changes in their symptoms, their satisfaction with treatment, and the degree to which they perceive their disease to affect their quality of life. Each scale is transformed to a score of 0 to 100, where higher scores indicate better function (e.g. less physical limitation, less angina, and better quality of life).
(6) Incidence of cardiovascular event during the 12 weeks such as developing into unstable angina, acute myocardial infarction and even death.

### Safety outcomes

Safety outcomes include vital signs (temperature, heart rate, breathing, and blood pressure after 10 min of rest), coagulation function test, liver and renal function, routine blood tests, urine and stool tests, and electrocardiograph (ECG).

### Management and statistical analysis

Statistical analysis will be performed by the Drug Clinical Research Center of Guangzhou University of TCM, 2 independent trained data administrators will read the CRFs and record the data on the EpiData 3.1 software, the study monitor will cross-check the electronic case report forms against the data administrators’ records on the purpose of ensuring the accuracy and reliability of the data. The database will be locked after blinding state data review and the statistical analysis can be performed only with the main investigator, sponsor, and statistical analyst.

Statistical analysis will be performed by a statistician blinded to the whole trial process using SAS 9.3 statistical software packages. The full analysis set (FAS) is the primary analysis set, in which one group receives NXT capsule treatment and the other placebo treatment. For the evaluation of curative effect in this trial, the per-protocol set (PPS) was used. Efficacy assessment will be performed through FAS and PPS. Safety evaluation will also be conducted. Continuous variables will be described using means and standard deviations and tested with Student’s *t* tests. Categorical variables are expressed in frequency counts and percentages and tested with chi-squared tests. More details will be described in a formal statistical analysis plan. Baseline balance between groups will be performed by a chi-square test or analysis of covariance (ANOVA). All collected data will be processed by professional statisticians using SAS 9.3 software; a two-sided *P* value of < 0.05 is considered statistically significant.

## Discussion

This study is a randomized, double-blind, placebo-controlled trial designed for NXT capsule to evaluate the efficacy and safety in the treatment of CSA. Coronary heart disease is the leading cause of death worldwide, the financial impact of treating CHD and angina can be large [15, 16]. According to World Health Organization statistics, CHD accounts for 17.3 million deaths/year, the annual global CVD mortality could be rising to 23.6 million by 2030 [17]. CSA affects a large population across the world and thus has a high mortality rate [18, 19]. This study focus on the improvement of participants’ symptoms, it is meaningful in the evaluation of the efficacy on CSA [20]. TCM has a considerable role in alleviating symptoms and maximizing quality of life of CSA patients. Hence, the efficacy of angina pectoris symptoms and TCM syndromes will be selected as therapeutic indicators, since they are correlated to patients’ symptoms and quality of life.

NXT Capsule, a modern patent traditional Chinese medicine, is mainly made up of Huangqi, Danshen, Quanxie, Shuizhi, and other sixteen herbs. It is extensively used to treat coronary heart disease, stroke, and other cardiovascular and cerebrovascular diseases, with the activity of anti-coagulation, anti-inflammatory, protecting endothelial cells, anti-atherogenic and plaque stabilization. Previous studies have shown that NXT capsule is safe and effective in cardiovascular protection. NXT capsule is known as a potent adjuvant treatment for CSA [21–23]. On the purpose of determining the efficacy and safety of NXT capsule combined with conventional therapy for patients with CSA, we designed this multicenter, double-blind, placebo-controlled randomized clinical trial in accordance with the Consolidated Standards of Reporting Trials (CONSORT) guidelines and the “One study, one primary outcome” clinical trial methodology [24]. We aim to provide new and high-quality evidence in NXT capsule treatment for patients with CSA. Furthermore, this protocol presents a detailed and practical methodology for future clinical trials of developing TCM.

## Trial status

The recruitment date is from2021/05/13 To 2023/07/17. Currently, patient recruitment for the trial is on-going.

## Data Availability

All data will be made available.

## Abbreviations

AEs: Adverse events
CSA: Chronic stable angina
CHD: Coronary heart disease
CONSORT: Consolidated Standards of Reporting Trials
CSS: Canadian Cardiovascular Society
CPMs: Chinese patent medicines
CFDA: China Food and Drug Administration
ECG: Electrocardiograph
FAS: Full analysis set
PPS: Per-protocol set
PCI: Percutaneous coronary intervention
SAQ: Seattle Angina Questionnaire
TCM: Traditional Chinese medicine
NXT: Naoxintong
